# Problems With GO Enrichment as an Endpoint for Ecological Omics Interpretation

**DOI:** 10.1111/mec.70570

**Published:** 2026-10-03

**Authors:** Ehsan Pashay Ahi, Nidal Karagic, Spiros Papakostas

**Affiliations:** ^1^ Organismal and Evolutionary Biology Research Programme, Faculty of Biological and Environmental Sciences University of Helsinki Helsinki Finland; ^2^ Institute of Biotechnology, Helsinki Institute of Life Science (HiLIFE), University of Helsinki Helsinki Finland; ^3^ Department of Science and Technology International Hellenic University Thessaloniki Greece

**Keywords:** ecological omics, gene ontology (GO) enrichment, GO‐deep integrative framework, mechanistic interpretation, non‐model organisms

## Abstract

Gene Ontology (GO) enrichment analysis has been widely adopted for interpretating omics data, including in ecological research. Although valuable for summarizing large gene lists into functional categories, it is often treated as the final interpretive step. In ecological omics, where functional annotations are frequently incomplete and experimental validation is often difficult, this reliance can lead to repetitive, generic conclusions that lack environmental and mechanistic specificity. Although previous literature has addressed the statistical and annotation‐related limitations of GO enrichment, its role in shaping the broader interpretive process has received less critical attention. Here, we highlight a conceptual weakness: the treatment of enriched GO terms as definitive biological insight. We focus particularly on ecological studies of non‐model organisms in complex, and heterogeneous environments. We discuss complementary strategies, including co‐expression network analysis, integration with environmental metadata, phylogenetically informed annotation, and AI‐assisted functional inference. To promote more context‐sensitive interpretation, we introduce the concept of *GO‐Deep*, which frames GO enrichment as a starting point for integrative, multilayered functional insight. By reframing GO enrichment as an entry point rather than a conclusion, ecological omics can move toward more mechanistically informative and environmentally grounded interpretations of high‐throughput data.

## 1 Introduction

Since the emergence of high‐throughput sequencing and transcriptomic profiling, functional enrichment analysis has become a foundational step in interpreting large‐scale omics data (Carbon et al. 2019; Reimand et al. 2019). Among available approaches, Gene Ontology (GO) enrichment analysis has been widely adopted due to its accessibility, standardized vocabulary, and ability to condense complex gene lists into biologically interpretable terms (Das et al. 2020; Ryan et al. 2025). Originally developed as a structured framework for annotating genes across species in terms of molecular function, cellular component, and biological process, GO was not intended to serve as an endpoint in biological reasoning (Gaudet and Dessimoz 2017). In practice, however, it is increasingly treated as such, particularly in studies in which direct experimental validation is limited or infeasible (key concepts used throughout the article are defined in Box 1).

BOX 1 **Glossary of key concepts used in this article.**

*Gene Ontology (GO)*: A structured vocabulary used to classify gene functions into biological process, molecular function, and cellular component categories across species.

*GO enrichment analysis*: A statistical method used to identify GO terms that are overrepresented in a subset of genes compared to a background set, often used to interpret omics data.

*Overrepresentation analysis (ORA)*: A type of enrichment analysis that determines whether certain terms occur more often than expected by chance within a given gene list.

*Functional annotation*: The process of assigning biological meaning to gene or protein sequences, often based on homology or curated databases like GO.

*Semantic generalization*: The tendency of GO terms to represent broad functional categories that may not reflect specific biological mechanisms or ecological roles.

*Homology‐based annotation*: The inference of gene function in poorly studied species by comparing their sequences with those of genes with known functions in model organisms.

*Context‐specific interpretation*: The consideration of environmental, developmental, or species‐specific factors when analysing gene function or expression patterns.

*Environmental metadata*: Associated data describing the physical or chemical conditions (e.g., temperature, pH, salinity) under which biological samples were collected.

*Semantic saturation*: The repeated reporting of similar GO terms across unrelated studies, leading to reduced novelty and indistinct conclusions in the literature.

*Interactome mapping*: The construction of protein–protein interaction networks that help to reveal relationships and potential functional groupings among gene products.

*Co‐expression networks*: Gene networks based on shared expression patterns across conditions or samples, used to identify functionally coordinated modules.

*AI‐assisted functional inference*: The use of machine learning or language‐based models to predict gene functions beyond those available through standard annotation resources.

*Environment Ontology (EnvO)*: A controlled vocabulary used to describe environmental features and conditions, allowing integration with genomic and functional data.

*Annotation bias*: Uneven distribution of gene function annotations across species or gene types, which can skew the results of enrichment analyses.

*Phylogenetically informed annotation*: The assignment of gene functions using evolutionary relationships, to improve specificity in non‐model species.

*Multi‐omics integration*: The combination of different omics layers (e.g., transcriptomics, proteomics, metabolomics) to provide a more comprehensive view of biological function.

*Temporal modelling*: Analytical approaches that incorporate time‐course data to identify dynamic changes in gene activity and regulatory interactions.

*Mechanistic hypothesis*: A testable explanation of how molecular components drive biological processes under specific conditions, beyond descriptive summaries.

*Interpretive endpoint*: The final step in a data analysis pipeline at which biological meaning is assigned; in this article, the critique focuses on the use of GO enrichment as such an endpoint.

The routine incorporation of GO enrichment into omics workflows has encouraged a pattern in which enriched GO terms are frequently interpreted as conclusive representations of underlying biology, often without deeper mechanistic interrogation (Gaudet and Dessimoz 2017; Timmons et al. 2015; Tomczak et al. 2018). This tendency is especially evident in ecological and environmental genomics, where functional interpretation is constrained by incomplete annotation resources, the lack of tractable experimental systems, and substantial biological variability across environments and taxa (Chalifa‐Caspi 2021; Freedman and Sackton 2024; Fridrich et al. 2019; Mora‐Márquez et al. 2021; Primmer et al. 2013; Tomczak et al. 2018; Vandepoele et al. 2024). In these contexts, GO enrichment may become both the analytical and narrative endpoint, with studies concluding that processes such as “oxidative stress response,” “immune response”, or “signal transduction” are involved solely because their associated GO terms are overrepresented among differentially expressed genes (Ayllon‐Benitez et al. 2020; Mubeen et al. 2022; Wei et al. 2020; Wijesooriya et al. 2022). In some cases, enrichment results may even implicate biological processes associated with anatomical structures absent from the target species because annotations were transferred from model organisms such as mice or humans (Haynes et al. 2018; Oziolor et al. 2021; Wei et al. 2020).

Although GO enrichment provides a rapid overview of broad functional trends, it also introduces important interpretive risks. GO terms are inherently hierarchical and generalized, and enrichment reflects statistical overrepresentation rather than mechanistic specificity or causal relevance. Consequently, reliance on GO enrichment alone may create a misleading sense of biological resolution, especially when context‐specific factors such as environmental conditions, species‐specific pathways, or gene‐regulatory dynamics are not integrated (Ahi and Singh 2024; Fulcher et al. 2021; Khatri et al. 2012; Kotlyar et al. 2022; Musella et al. 2025; Song et al. 2025; Tomczak et al. 2018; Tran et al. 2019; Young et al. 2010; Zehr et al. 2025; Zhao and Rhee 2023). In this way, annotation‐driven interpretation can gradually shift into a heuristic shortcut that obscures more complex, context‐dependent, or potentially novel biological mechanisms.

The widespread use of GO enrichment has also contributed to increased redundancy in published interpretations (Ballouz et al. 2017; Chen et al. 2022; Gu and Hübschmann 2023; Ozisik et al. 2022; Stoney et al. 2018; Wijesooriya et al. 2022; Y. Zhao et al. 2020). Across diverse fields ranging from microbial ecology to plant environmental biology, similar sets of GO terms are repeatedly reported, regardless of species, ecosystem or stressor. While some recurrence may reflect genuinely conserved biological responses, part of this convergence may also arise from the limited granularity of GO categories and the homology‐driven nature of many annotations, particularly in non‐model organisms (Aleksander et al. 2023; Bhatia et al. 2024; Bianca et al. 2023; Carbon et al. 2017; Tomczak et al. 2018). Consequently, conclusions drawn from GO enrichment alone may fail to differentiate between studies in a mechanistically meaningful way.

Although numerous studies have highlighted statistical and methodological limitations of GO enrichment, including inappropriate background gene sets, overreliance on *p*‐values, and redundancy among enriched terms (Chicco and Agapito 2022; Koopmans 2024; Wijesooriya et al. 2022; K. Zhao and Rhee 2023; Ziemann et al. 2024), comparatively less attention has been paid to the conceptual implications of treating GO enrichment as a conclusive interpretive endpoint. This distinction is critical, as it shifts the question from whether GO analysis is statistically correct to whether it is biologically sufficient to address ecological or mechanistic questions. This issue is particularly pronounced in ecological omics, where gene functions are often poorly characterized, and validation pipelines remain limited (Freedman and Sackton 2024; May et al. 2025; Primmer et al. 2013). Under these conditions, reliance on GO enrichment may reinforce oversimplified narratives, encourage repetitive interpretations, and potentially delay the identification of system‐specific or previously unrecognized mechanisms. Recognizing the limitations of GO enrichment as an interpretative endpoint is therefore essential if ecological functional genomics is to move beyond annotation and toward explanation. Key recurring interpretative risks that contribute to generic or misleading interpretations are summarized in Table 1. Importantly, this critique does not dismiss GO enrichment as a useful analytical approach. GO enrichment can be entirely appropriate, and in some cases sufficient, for summarizing gene lists, generating preliminary hypotheses, comparing broad functional patterns, or assessing conserved biological programs. The concern arises when results are used to infer causal mechanisms, ecological function, cell‐type specificity, or adaptive significance without additional contextual or experimental evidence.

**TABLE 1:** Common interpretive failure modes of GO enrichment in ecological omics.

Failure mode	Typical symptom in ecological omics papers	What drives it	Practical mitigation (brief)	Key references
Broad/Umbrella terms mistaken for mechanism	“response to stress/Immune response” used as final biological story	GO terms are semantic labels, not mechanistic models	Follow‐up with networks, time structure, or trait links	Gaudet and Dessimoz (2017), Stanford et al. (2020), Khatri et al. (2012), Zhao and Rhee (2023)
Annotation bias/Uneven coverage	Same well‐annotated processes dominate across studies	Better‐studied genes/Species get richer GO	Check annotation depth; use lineage‐aware annotation; report coverage	Haynes et al. (2018), Oziolor et al. (2021), Xue and Rhee (2023), Primmer et al. (2013)
Ontology/Annotation drift over time	Old conclusions no longer reproduce under newer GO releases	GO structure + annotations change	Report GO/Annotation versions; sensitivity checks	Tomczak et al. (2018), Jacobson et al. (2018), Aleksander et al. (2023)
Selection bias in RNA‐seq enrichment	Bias toward long/Highly expressed genes	DE detectability affects gene lists	Use bias‐aware methods or corrections	Young et al. (2010), Timmons et al. (2015)
Redundant term inflation	Long lists of near‐duplicate GO terms	GO hierarchy produces overlapping categories	Reduce redundancy (Clustering/Trimming/Filtering)	Jantzen et al. (2011), Gu and Hübschmann (2023), Ozisik et al. (2022), Wang et al. (2020)
Gene‐set overlap/Inter‐gene correlation ignored	Overconfident *p*‐values; overlapping pathways “all significant”	Shared genes & correlation violate assumptions	Use overlap‐aware/Correlation‐aware tests	Tarca et al. (2012), Wu and Smyth (2012), Simillion et al. (2017)
“*p* < 0.05” as the decision rule	Enrichment becomes a binary story; weak/Unstable terms overinterpreted	Thresholding + multiple testing + small gene sets	Emphasize effect size, stability, calibration	Wijesooriya et al. (2022), Li et al. (2021), Ziemann et al. (2024), Chicco and Agapito (2022)
Semantic saturation/Repeated narratives across ecosystems	Different taxa/Stressors report the same GO “hits”	Conserved stress programs + broad vocabulary	Require context linkage (Environment/Traits/Time)	Ballouz et al. (2017), Tomczak et al. (2018), Zhao et al. (2020)

*Note:* Common ways GO enrichment is overinterpreted when used as a narrative endpoint in ecological and environmental omics. Each row describes a recurring failure mode, its typical symptom in published studies, the underlying driver (e.g., annotation bias, redundancy, selection bias), and a practical mitigation. The final column lists key methodological and conceptual references supporting each issue.

## 2 Echoes Across Ecosystems: Recurring GO Interpretations Across Disparate Systems

Across ecological omics studies, a recurring concern is that GO enrichment analyses frequently converge on broad biological process terms across different species, stressors, or environmental contexts, although the extent of this pattern remains to be systematically quantified in ecology (Domeniconi et al. 2016; Forés‐Martos et al. 2021; Hartmann et al. 2022; Quinlan et al. 2025; Wang et al. 2022; C. Zhao and Wang 2018). This pattern is particularly striking when transcriptomic or proteomic datasets from unrelated organisms, ranging from plants and corals to fungi and soil microbes, report the same or similar enriched GO terms (Djeddi et al. 2025; Fattel et al. 2022; Song et al. 2025; Sosa et al. 2023; Tian et al. 2022; Xue et al. 2023). Common outputs such as “response to oxidative stress,” “signal transduction”, or “defence response” are frequently presented as final interpretations, despite being observed in highly disparate ecological settings. For instance, in studies of thermal stress in reef‐building corals, oxidative stress‐related GO terms are routinely overrepresented among upregulated genes (Aguilar et al. 2024; Nunn et al. 2025; Yuyama et al. 2022). Similar patterns have been reported in drought‐stressed crops such as maize and rice, where comparable GO terms appear despite differences in tissue type, species physiology, and exposure scale (Gillani et al. 2022; Y. Li et al. 2024; Liang et al. 2021; S. Liu et al. 2021; Lu et al. 2022). In fungal systems exposed to metal‐contaminated soils, enrichment of categories such as “metabolic process” is often observed (Dai et al. 2023; Liu et al. 2022; Shi et al. 2022). Likewise, in microbial community responses to oil spills or eutrophication, GO terms related to “catabolic process” and “transport” repeatedly emerge (Chen et al. 2023; Peng et al. 2024; Xu et al. 2022; Zhao et al. 2023). Although these recurring outcomes are not necessarily incorrect in a statistical sense, their ubiquity raises questions about their specificity, resolution, and ecological informativeness.

The tendency for similar GO terms to appear across unrelated studies can be partly explained by two factors: the generality of the GO vocabulary and the evolutionary conservation of core stress responses (Burton et al. 2021; Koc and Caetano‐Anolles 2017; Murray and Bergland 2025; Tomczak et al. 2018; S. Wang et al. 2022; Wu, Goh, et al. 2021). Certain biological processes, such as redox homeostasis or protein folding, are fundamental to cellular life and are therefore activated under a wide range of environmental stressors across diverse taxa (Erdős et al. 2019; Kidd et al. 2023). While part of this recurrence likely reflects genuinely conserved biological responses shared across taxa, these general responses can obscure more specific and ecologically meaningful findings when they dominate the functional summary of a dataset. In effect, such patterns risk flattening biological interpretation into a limited set of recurring functional narratives that do not meaningfully distinguish one ecological system from another (Gaudet and Dessimoz 2017; Jantzen et al. 2011; Thomas 2017; Tomczak et al. 2018; Y. Zhao et al. 2020). In some cases, authors attempt to contextualize enriched GO terms by referring to prior literature in similar organisms or environments. However, this can result in circular reasoning, where previous findings based on the same generic GO terms are used to justify current interpretations (Gillis and Pavlidis 2013; Jacobson et al. 2018; Richardson et al. 2024; Saxena et al. 2022; Y. Zhou et al. 2019). Without deeper molecular detail or contextual inference, this recursive pattern may foster what we refer to here as semantic saturation: the repeated use of broad functional labels that can create the appearance of biological insight without necessarily advancing new hypotheses or mechanistic understanding (Phan et al. 2025; R. Richardson et al. 2024; Saxena et al. 2022; Valverde et al. 2025). This pattern is particularly problematic in comparative or meta‐analytical studies, in which similarity in GO enrichment profiles may be used to infer shared biological strategies or conserved ecological responses (Altenhoff, Vesztrocy, et al. 2024; Mubeen et al. 2022; Tomczak et al. 2018). Unless such similarity is supported by independent lines of evidence, such as phylogenetic relatedness, shared regulatory motifs, or comparable environmental parameters, reliance on GO enrichment as the primary evidence of functional convergence remains tenuous (Ovens et al. 2021; Valverde et al. 2025; Wijesooriya et al. 2022). Moving beyond this echo chamber of generic terms will likely require broader integration of context‐specific metadata, orthogonal validation tools and methods that can identify more targeted molecular patterns (Altenhoff, Vesztrocy, et al. 2024; Bourgeais et al. 2021; Edera et al. 2022; R. Richardson et al. 2024; Saxena et al. 2022; Verschaffelt et al. 2021). Systematic text‐mining analyses or meta‐analytic assessments of recurring GO terms across ecological omics studies would be valuable for evaluating the prevalence, taxonomic distribution, and ecological specificity of this phenomenon. Until such analyses are performed, many omics studies may continue to converge on broadly similar functional interpretations despite involving organisms and ecosystems with fundamentally different dynamics.

## 3 The Limits of Semantics: Why Broad GO Terms Often Fall Short of Explaining Ecological Mechanisms

The GO framework was developed to provide a shared vocabulary for annotating gene functions across species through a hierarchical structure that ranges from broad categories to more detailed terms (Aleksander et al. 2023; Carbon et al. 2017). While this architecture is highly valuable for functional classification and cross‐species comparisons, it imposes semantic constraints that can limit interpretation in ecological systems (Gaudet and Dessimoz 2017). GO terms, especially those associated with biological processes, are designed to capture general aspects of cellular or physiological activity rather than specify how, when, or why those processes occur within an environmental or evolutionary context (Gaudet and Dessimoz 2017; Lofeu and Ahi 2026; Pavey et al. 2012; Stanford et al. 2020). This level of generality is not inherently problematic when the objective is broad functional classification or descriptive summarization. However, it becomes limiting when broad GO categories are interpreted as mechanistic explanations for ecological responses without additional evidence linking the enriched terms to the relevant organismal, environmental, temporal, or regulatory context. In molecular ecology, where a central goal is often to understand how organisms interact with their environment or adapt to ecological pressures, broad GO terms frequently lack the specificity needed for mechanistic interpretation (Stanford et al. 2020). A common example is enrichment of the term ‘response to stress,’ which may encompass diverse cellular defence pathways, RNA‐mediated stress sensing, and cell‐state regulation, among other multifunctional processes (Ballouz et al. 2017; Nagaraj et al. 2026; Tomczak et al. 2018). While such findings indicate that a biological response is occurring, they do not identify the underlying trigger, clarify which pathways are driving the response, or explain how those molecular changes relate to ecological fitness or resilience (Khatri et al. 2012; Maleki et al. 2020; Stanford et al. 2020). This limitation is substantial because it constrains the development of mechanistically grounded models of organism‐environment interaction (Gudmunds et al. 2022).

Examples of this limitation occur across diverse ecological systems. In plants exposed to salt stress, enrichment of terms such as “ion transport” or “response to osmotic stress” may indicate broad cellular adjustment, but often fails to distinguish among early signalling cascades, hormone‐mediated regulation, or downstream growth inhibition (Seifikalhor et al. 2019; Xiao and Zhou 2023; H. Zhou et al. 2024). In fungal systems, such as wood‐decaying basidiomycetes, enrichment of “metabolic process” provides little insight into whether the observed metabolic shift reflects lignin degradation, carbon limitation, or secondary metabolite synthesis (Gaskell et al. 2016; Riley et al. 2014). Similarly, in coastal and estuarine metatranscriptomes from polluted waters, enrichment of umbrella terms such as “response to toxin” or “transmembrane transport” may be statistically robust while remaining functionally opaque because of the diversity of compounds, taxa, and ecological interactions involved (Cerro‐Gálvez et al. 2021; DeLeo et al. 2021; Knapik et al. 2020).

Animal systems are not immune to this limitation. In invertebrates like bivalves or crustaceans, exposure to fluctuating oxygen levels often yields GO terms such as “response to hypoxia” or “mitochondrial activity,” yet these categories provide limited resolution regarding the type of stress sensors involved, the role of reactive oxygen species, or whether apoptotic or repair pathways are activated (Adzigbli et al. 2024; Montúfar‐Romero et al. 2024; Rathburn et al. 2013). Likewise, in vertebrate systems such as amphibians exposed to environmental stressors, including acidified larval habitats or pathogen challenge, broad categories such as “immune system process” may conflate innate and adaptive immune components while providing little information about tissue‐specific or temporal dynamics (Campbell et al. 2018; Krynak et al. 2015; Price et al. 2015).

Part of this limitation arises from the annotation process itself (Ekblom and Wolf 2014; Hart et al. 2020; Jones et al. 2014). For many non‐model organisms, gene annotation is inferred through homology to better‐characterized species. As a result, annotations tend to emphasize well‐conserved functions while underrepresenting more specialized roles, such as lineage‐ or habitat‐specific biology (Ekblom and Wolf 2014; Hart et al. 2020; Jones et al. 2014; Vlasova et al. 2021). This reinforces the use of general terms and limits the potential for GO analysis to detect contextually important, but less evolutionarily conserved, mechanisms, including adaptive regulatory variation generated through alternative splicing (Cassan et al. 2021; Haynes et al. 2018; Singh and Ahi 2022; Singh et al. 2026). Moreover, the design of enrichment tools often prioritizes statistical over semantic precision (Bakulin et al. 2024; Fulcher et al. 2021; Khatri et al. 2012; Maleki et al. 2020; Young et al. 2010). Many algorithms report significant enrichment for GO terms regardless of their interpretive clarity or redundancy (Wu, Hu, et al. 2021; K. Zhao and Rhee 2023). In ecological studies, where the biological questions are often embedded in complex systems involving multiple stressors, trophic levels, and habitat types, such ambiguity undermines the ability to generate mechanistically meaningful hypotheses (Stanford et al. 2020). To move beyond the constraints of semantic generalization, functional interpretation in ecology will require strategies that pair GO enrichment with additional layers of data and analytical refinement. These may include the integration of environmental metadata, time‐series expression patterns, or inferred regulatory networks that help clarify when and how a given process is engaged (Amar et al. 2021; Cernava et al. 2022; Morabito et al. 2023; Oh and Li 2021; Van den Broeck et al. 2020). Without such integration, GO‐based interpretations may remain functionally shallow even when statistically robust.

## 4 An Under‐Examined Weakness: Beyond the Known Pitfalls of GO Enrichment

The limitations of GO enrichment have been acknowledged in several methodological publications, with critiques typically focusing on statistical or structural issues (Maleki et al. 2020; Wijesooriya et al. 2022; Ziemann et al. 2024). These include improper use of background gene sets (Wijesooriya et al. 2022; Ziemann et al. 2024), failure to correct for multiple testing (Wijesooriya et al. 2022), term redundancy (Gu and Hübschmann 2023; Wang et al. 2020) and biases introduced by uneven annotation coverage across species (Haynes et al. 2018; Jacobson et al. 2018; Tomczak et al. 2018). While such concerns are important and merit ongoing attention, they represent only part of a larger interpretive issue that has received relatively little scrutiny: the tendency to accept enriched GO terms as standalone biological conclusions rather than as intermediate summaries requiring contextual validation (Fulcher et al. 2021; Maleki et al. 2020; K. Zhao and Rhee 2023). In many cases, studies that highlight GO enrichment results provide limited discussion of what those terms mean physiologically or ecologically (Stanford et al. 2020; Wijesooriya et al. 2022). The presence of statistically significant enrichment is often treated as synonymous with a mechanistic explanation, despite the fact that enrichment establishes only a statistical association between a gene set and a functional category (Fulcher et al. 2021; Geistlinger et al. 2020; Maleki et al. 2020; K. Zhao and Rhee 2023). This pattern reflects a deeper conceptual weakness that has not been extensively discussed in the literature: the misinterpretation of annotation output as biological insight, particularly in fields where downstream experimentation is difficult to perform (Stanford et al. 2020).

Although technical critiques have improved awareness of best practices in GO analysis, they have not challenged the core assumption that enrichment alone provides sufficient interpretive depth (Buzzao et al. 2024; Maleki et al. 2020; Wijesooriya et al. 2022). As a result, the literature continues to reflect a gap between what enrichment tools can detect and the conclusions that are sometimes drawn from them (Buzzao et al. 2024; Wijesooriya et al. 2022). In ecology, where functional characterization tools are limited, this gap is often wider, because the lack of alternative interpretive frameworks amplifies reliance on GO as the primary, and sometimes the only, means of assigning biological meaning to omics data (Gudmunds et al. 2022; Stanford et al. 2020; Y. Wang et al. 2023; B. Xue and Rhee 2023). Furthermore, even comprehensive assessments of GO usage in bioinformatics have largely overlooked the broader issue of interpretive dependence. Large‐scale surveys of enrichment misuse have focused mainly on reproducibility and reporting (e.g., background lists, incomplete methods) and on the misuse of statistical thresholds (Wijesooriya et al. 2022). Similarly, benchmarking studies emphasize method variability and calibration rather than the narrative framing of enrichment results as biological endpoints (Buzzao et al. 2024; Geistlinger et al. 2020). Few studies have evaluated how GO outputs are presented as final biological conclusions in Results and Discussion sections (Buzzao et al. 2024; Wijesooriya et al. 2022). A practical reporting and interpretation checklist tailored to ecological omics is provided in Table 2. This under‐examined weakness is not limited to any one taxonomic group or study type. In fungal saprotrophs, enriched terms like “cell wall organization or biogenesis” are frequently reported in wood‐decay experiments and then linked, sometimes indirectly, to colonization strategy or substrate preference (Kowalczyk et al. 2019; Mäkinen et al. 2019). In insects, GO terms such as “response to pheromone” are sometimes linked to behavioural hypotheses, yet these terms alone do not establish population‐level behaviour without direct functional validation (Deanhardt et al. 2023; Doyle et al. 2022; Marshall et al. 2020). Such inferences are not necessarily statistically incorrect, but they exceed what can be inferred from functional categories without contextual evidence and targeted experiments (Fulcher et al. 2021; Geistlinger et al. 2020; Maleki et al. 2020; K. Zhao and Rhee 2023). Our critique is therefore directed at a recurring interpretive practice rather than at individual studies. Because the adequacy of a GO‐based conclusion depends on the study question, annotation resources, and feasibility of validation, later mechanistic refinement should not automatically be interpreted as evidence that earlier GO‐based interpretations were erroneous. Addressing this issue will require interpretive standards that move beyond statistical thresholds and explicitly ask whether an enriched GO term advances biological understanding in its ecological context (Buzzao et al. 2024; Li et al. 2021; Stanford et al. 2020; Wijesooriya et al. 2022; K. Zhao and Rhee 2023).

**TABLE 2:** Reporting checklist for GO enrichment in ecological omics.

Checklist item to report	Why it matters	Minimum recommended practice	Key references
Background gene universe	Wrong background inflates terms	Define and justify universe used	Wijesooriya et al. (2022), Ziemann et al. (2024)
GO/Annotation versions	Conclusions change with GO updates	Report GO release + annotation source/Date	Tomczak et al. (2018), Jacobson et al. (2018), Aleksander et al. (2023)
Bias and detectability controls	RNA‐seq selection bias	Apply bias correction/Acknowledge	Young et al. (2010), Timmons et al. (2015)
Redundancy handling	Long redundant term lists mislead	Trimming/Clustering with rationale	Jantzen et al. (2011), Gu and Hübschmann (2023), Ozisik et al. (2022)
Multiple testing + thresholding	False positives & “binary” narratives	Control FDR; report effect sizes/Stability	Wijesooriya et al. (2022), Li et al. (2021), Chicco and Agapito (2022)
Overclaiming mechanism from enrichment	GO ≠ causality	Explicitly label enrichment as “hypothesis‐generating”	Gaudet and Dessimoz (2017), Stanford et al. (2020), Zhao and Rhee (2023)
Annotation depth across taxa	Cross‐species comparisons confounded	Report coverage and mapping rates	Haynes et al. (2018), Oziolor et al. (2021), Xue and Rhee (2023)
Share gene lists + code	Reproducibility	Publish inputs/Outputs & scripts	Wijesooriya et al. (2022), Geistlinger et al. (2020), Reimand et al. (2019)

*Note:* Recommended reporting and interpretation items to improve transparency, reproducibility, and appropriate framing of GO enrichment in ecological omics. Items cover background gene universe specification, versioning of GO/annotations, bias and redundancy handling, multiple testing and stability, annotation depth across taxa, and sharing of inputs/code. References highlight community guidelines, methodological critiques, and benchmarking studies motivating each checklist item.

## 5 Why Ecology is Especially Vulnerable: Complexity, Inference, and the Mirage of Function

The field of ecology may be particularly vulnerable to the interpretive pitfalls associated with overreliance on GO enrichment (Reimand et al. 2019; Stanford et al. 2020). This vulnerability does not stem from a lack of analytical sophistication, but rather from the inherent complexity of ecological systems and the current limitations of functional annotation for non‐model organisms (Gudmunds et al. 2022; Haynes et al. 2018; Wood et al. 2019; B. Xue and Rhee 2023). In many ecological applications, omics approaches are applied to species with poorly characterized genomes, under field conditions that cannot be easily replicated, and across systems shaped by numerous interacting biotic and abiotic variables (Fulcher et al. 2021; Jackson et al. 2021; Pirotta et al. 2022; Stanford et al. 2020). These challenges make statistical summaries of function particularly attractive, but they also increase the risk that those summaries will be accepted without sufficient biological justification (Fulcher et al. 2021; Reimand et al. 2019).

One reason GO enrichment is heavily relied upon in ecological studies is the scarcity of experimentally validated gene‐function data (Gudmunds et al. 2022; Haynes et al. 2018; R. Richardson et al. 2024; B. Xue and Rhee 2023). Unlike biomedical research, where model organisms and perturbation experiments often permit direct testing of functional hypotheses, ecological genomics frequently operates with limited capacity to connect genes to phenotypes (Gudmunds et al. 2022; Waldvogel et al. 2020). For many plants, fungi, and animals living in complex environments, annotations are assigned primarily through sequence homology, which can overlook or misrepresent context‐specific functions (Ejigu and Jung 2020; Haynes et al. 2018; Sinha et al. 2020). Microbial communities present additional challenges because functional inference often depends on metagenomic binning and probabilistic gene assignment (Han et al. 2025; Mallawaarachchi et al. 2024; Meyer et al. 2022). The resulting annotation landscape is therefore both incomplete and uncertain, encouraging greater reliance on enrichment tools that appear to provide interpretive clarity (Haynes et al. 2018; Reimand et al. 2019; Wood et al. 2019).

Environmental complexity adds another layer of difficulty. In ecology, omics studies are rarely conducted under single‐variable conditions (Jackson et al. 2021; Pirotta et al. 2022). Instead, organisms are exposed to combinations of temperature changes, nutrient fluctuations, pathogens, chemical pollutants, and interspecies interactions (Jackson et al. 2021; Pirotta et al. 2022). GO enrichment is poorly suited to disentangling such overlapping stimuli, as it does not capture regulatory timing, synergistic effects, state‐dependent post‐transcriptional regulation, or indirect interactions (Ahi 2026; Aleksander et al. 2023; Reimand et al. 2019; Thomas et al. 2019). For example, in Sphagnum‐microbiome systems under warming, metatranscriptomes show microbial shifts that induce plant heat‐shock responses; these signals could map to broad GO categories without revealing causes (Carrell et al. 2022; Kolton et al. 2022). In such cases, enriched terms like “response to stimulus” or “metabolic process” offer little clarification (Reimand et al. 2019; Stanford et al. 2020).

Another factor contributing to vulnerability in ecology is the immense diversity of species, life histories, and ecological contexts that may be compressed into similar GO categories (Altenhoff, Nevers, et al. 2024; Nevers et al. 2022; Oziolor et al. 2021). Insect species occupying distinct ecological niches may possess divergent physiological pathways even when GO analyses recover similar categories such as “cuticle development” or “pheromone signalling” (Cao et al. 2023; Holze et al. 2020; Vernier et al. 2019; Yang et al. 2024). Likewise, forest soil fungi from contrasting biomes may show enrichment of broad categories such as “oxidoreductase activity,” while utilizing entirely different substrates and ecological niches (Auer et al. 2024; Smith et al. 2024; Zavarzina et al. 2018). Such cases illustrate how similar GO terms can mask substantial ecological and mechanistic differences, challenging the assumption that GO term similarity necessarily implies functional equivalence across systems (Altenhoff, Nevers, et al. 2024; Nevers et al. 2022; Oziolor et al. 2021).

Because direct manipulation of ecological systems is often infeasible or ethically constrained, the conclusions of many ecological omics studies depend heavily on the interpretation of functional annotations (Gudmunds et al. 2022; Stanford et al. 2020). For this reason, downstream validation should be scaled to the study system. In many field‐based or non‐model studies, improved annotation, redundancy control, environmental linkage, and network‐based prioritization may represent the most realistic and informative core strategies, whereas experimental perturbation is more feasible in tractable or well‐resourced systems. In this setting, GO enrichment can produce a mirage of insight, giving the impression that function has been explained when, in fact, it has only been labelled (Reimand et al. 2019; Timmons et al. 2015). This is particularly problematic when such results are used to inform conservation strategies, management interventions, or evolutionary models (Hohenlohe et al. 2021; Shafer et al. 2015; Supple and Shapiro 2018).

If omics approaches are to contribute more meaningfully to ecological theory and practice, interpretive frameworks will need to recognize the limits of GO enrichment as a universal translator of function (Aleksander et al. 2023; Reimand et al. 2019). This may involve greater integration of environmental metadata (Eloe‐Fadrosh et al. 2024), broader use of environmental RNA data, including classes beyond mRNA (Ahi and Schenekar 2025), comparative regulatory analyses such as gene regulatory network inference, and co‐expression network analysis (Aibar et al. 2017; Morabito et al. 2023; Skok Gibbs et al. 2022) and incorporation of trait‐based and ecosystem‐level observations (Moreira‐Saporiti et al. 2023). Without such approaches, there remains a substantial risk that functional interpretation in ecology will be shaped more by annotation convenience than by ecological relevance (Reimand et al. 2019; Stanford et al. 2020).

## 6 Breaking the Bottleneck: Integrative Strategies for Ecological Insight

The limitations of GO enrichment in ecological omics do not render the approach obsolete. Rather, they highlight the need for interpretative strategies that extend beyond enrichment statistics by incorporating biological, environmental, and network‐level context. Several complementary approaches can augment or refine GO‐based findings by providing additional biological and mechanistic resolution. These strategies are particularly valuable for studies involving non‐model organisms, heterogeneous environmental conditions, and systems where experimental manipulation is limited. Table 3 summarizes these approaches as classes of complementary strategies rather than as a mandatory analytical pipeline. The approaches differ substantially in feasibility, data requirements, and evidentiary strength. Some strategies, including redundancy reduction, co‐expression analysis, interactome mapping, and environmental metadata integration, can often be applied computationally to existing datasets. Others, such as time‐series multi‐omics, metaproteomics, stable‐isotope probing, synthetic communities, genome editing, and spatial or single‐cell assays, can provide deeper mechanistic insight but require additional sampling, infrastructure or experimentally tractable systems. AI‐assisted inference is treated here as a cross‐cutting tool for annotation refinement and hypothesis prioritization rather than as a standalone interpretive endpoint.

**TABLE 3:** Strategies to improve interpretation of ecological omics beyond GO enrichment.

Strategy	Main function	Tool/method	Ecological relevance	Strengths	Limitations	References
Co‐expression networks	Groups genes by shared expression profiles	WGCNA, MEGENA	Detects response modules in plants, fungi, animals	Captures condition‐specific responses	Sensitive to noise, batch structure, & confounding; co‐expression does not prove regulation or causality	Morabito et al. (2023), Ovens et al. (2021), Booth et al. (2024), Aci et al. (2024)
Interactome integration	Maps functional associations among proteins	STRING, Pathway Commons	Reveals coordinated pathways in microbial or animal systems	Can identify functional hubs	Prior‐knowledge & model‐organism bias; incomplete or orthology‐transferred networks for non‐models	Szklarczyk et al. (2023), Kotlyar et al. (2022), Mehryary et al. (2024), Ahi and Schenekar (2025)
Environmental metadata linkage	Connects gene function with measured habitat features	EnvO integration, correlation analysis	Contextualizes function in variable environments	Enhances ecological interpretation	Requires detailed metadata	Buttigieg et al. (2016), Cernava et al. (2022), Richardson et al. (2023), Vernette et al. (2022), Eloe‐Fadrosh et al. (2024)
AI‐based annotation refinement	Predicts functions using text or sequence embeddings	BioGPT, DeepGOPlus, Geneformer	Improves function calls in under‐annotated genomes	Species‐ or condition‐specific predictions	Training‐data bias toward well‐studied organisms; limited transparency in prediction logic; predictions require contextual validation	Luo et al. (2022), Kulmanov and Hoehndorf (2020), Kulmanov and Hoehndorf (2022), Theodoris et al. (2023), Gligorijević et al. (2021)
Phylogenetically informed annotation	Assigns function based on evolutionary context	eggNOG‐mapper with custom DBs	Improves function calls in plants, fungi, insects	Reduces misannotation	Requires curated phylogenies	Cantalapiedra et al. (2021), Altenhoff, Vesztrocy, et al. (2024), Altenhoff, Nevers, et al. (2024), Vandepoele et al. (2024), De Crécy‐Lagard et al. (2022)
Temporal modelling	Tracks changes over time or development	Dynamic Bayesian networks, pseudotime inference	Resolves phase‐specific responses in animals and plants	Adds directionality to interpretation	Requires time‐series data	Oh and Li (2021), Van den Berge et al. (2020), Suter et al. (2022), Marku and Pancaldi (2023)
Multi‐omics integration	Combines gene, protein, metabolite, and epigenetic data	MOFA+, mixOmics	Reveals causal relationships in complex systems	Provides a systems‐level view	High data demands, tool complexity	Ryan et al. (2025), Frese et al. (2024), Zhang et al. (2024), Li et al. (2024)

*Note:* Each strategy represents a complementary or downstream step that can enhance the resolution, contextual relevance, or mechanistic depth of functional analysis in diverse taxa and environments. Examples include co‐expression clustering, AI‐based annotation, and integration of environmental metadata.

### 6.1 Established and Emerging Computational Approaches

#### 6.1.1 Co‐Expression Networks and Interactome Mapping

GO functional enrichment alone provides limited insight into gene–gene relationships, even though these relationships are often central to ecological responses. Co‐expression approaches, such as WGCNA and its single‐cell extension hdWGCNA, group genes according to shared expression profiles and identify modules associated with environmental gradients or phenotypic traits (Aci et al. 2024; Booth et al. 2024; Duenser and Ahi 2026; Morabito et al. 2023). In plant‐microbe systems, co‐expression modules have revealed symbiotic or defensive responses that would not have been evident from GO enrichment alone (Niu et al. 2024; Pereira et al. 2024; Riaz et al. 2025). Protein–protein interaction networks and interactome resources, including STRING and context‐specific interactomes, provide a complementary layer of interpretation (Kotlyar et al. 2022; Mehryary et al. 2024; Szklarczyk et al. 2023). When integrated with GO enrichment results, these networks can help identify candidate regulators, pathway bottlenecks, and co‐regulated processes that may explain how a particular functional category becomes activated (Mehryary et al. 2024; Szklarczyk et al. 2023). At the same time, these approaches require cautious interpretation. Co‐expression does not demonstrate direct regulation or causality, and inferred modules may reflect underlying population structure, developmental stage, correlated environmental exposure, or technical batch effects rather than coordinated function. Likewise, interactome resources organize valuable prior knowledge, but their coverage and confidence scores depend heavily on experimental evidence, literature mining, orthology transfer, and database curation. The coverage of these resources is typically greatest for well‐studied organisms and substantially more limited for ecological non‐model taxa (Kotlyar et al. 2022; Mehryary et al. 2024; Pashay Ahi 2025; Szklarczyk et al. 2023). Nevertheless, when interpreted alongside environmental context, lineage information, and complementary evidence, co‐expression and interactome‐based approaches can extend functional interpretation substantially beyond what GO enrichment alone provides. Rather than serving as definitive proof of ecological mechanism, they are particularly valuable for prioritizing candidate pathways, identifying coordinated biological responses, and generating more mechanistically informed hypotheses for downstream investigation.

#### 6.1.2 Decoding Promiscuous Pathway Footprints

Promiscuous mediators and shared nodes make GO interpretation particularly challenging in ecology. Similar omics footprints can arise from different cues when Ca^2+^ decoders, MAPKs, hormone cross‐talk hubs, or two‐component systems are activated by many inputs; consequently, pathway attribution from membership alone is underdetermined (Ahi et al. 2025; Garcia‐Alonso et al. 2019; Holland et al. 2020; Schubert et al. 2018; Simillion et al. 2017; Tarca et al. 2012; Wiredja et al. 2017; D. Wu and Smyth 2012). Rather than treating this ambiguity as a dead end, pathway‐footprint and overlap‐aware approaches can help refine interpretation by asking whether the observed expression pattern is consistent with pathway activity, regulatory output, or recurring overlap among gene sets. For example, gene‐set overlap can inflate enrichment significance and increase redundancy among reported categories. Overlap‐aware approaches such as PADOG and SetRank, together with inter‐gene‐correlation corrections like CAMERA, can help mitigate this bias (Simillion et al. 2017; Tarca et al. 2012; D. Wu and Smyth 2012). Similarly, because gene membership alone does not necessarily indicate pathway activity, footprint‐based methods that infer pathway activation from downstream responsive genes or regulons, including PROGENy and DoRothEA coupled with VIPER, may provide greater specificity and tolerate pathway cross‐talk more effectively (Garcia‐Alonso et al. 2019; Holland et al. 2020; Schubert et al. 2018). Comparable principles apply to phosphoproteomics and single‐cell communication analyses. Kinase‐substrate relationships are frequently many‐to‐many, making substrate‐set approaches such as KSEA and PTM‐SEA potentially more informative than direct interpretation of individual phosphorylation events, although these approaches still require careful interpretation (Piersma et al. 2024; Wiredja et al. 2017). Likewise, ligand–receptor promiscuity in single‐cell communication analyses can artificially overconnect cell types unless biological priors or spatial constraints are incorporated (Pong et al. 2024; Su et al. 2022). Together, these approaches help move interpretation from broad category membership toward evidence of pathway activity, regulatory influence, and context‐specific signalling (Khersonsky and Tawfik 2010; Notebaart et al. 2018; Teschendorff and Enver 2017). They do not eliminate ambiguity, but they provide a practical route for reducing the semantic bottleneck of GO enrichment and generating more focused, testable ecological hypotheses.

#### 6.1.3 AI‐Assisted Annotation and Contextual Inference

Recent advances in language‐based models and deep learning have opened new avenues for supporting context‐sensitive functional inference. These models contribute to interpretation in different ways, and were not all developed specifically as gene‐function prediction tools. DeepGOPlus was designed for protein function prediction from sequence information (Kulmanov and Hoehndorf 2020), whereas broader models such as BioGPT and Geneformer can support literature mining, gene‐context representation, perturbation prediction, or hypothesis prioritization when integrated into appropriate downstream workflows (Luo et al. 2022; Theodoris et al. 2023). Related approaches also support functional inference through structural information (Gligorijević et al. 2021; Jiao et al. 2023), ontology‐aware prediction and zero‐shot learning (Kulmanov and Hoehndorf 2022), and literature‐ or knowledge‐graph‐derived relationships (Lim et al. 2025). For example, in arbuscular mycorrhizal systems, integrative transcriptomics and imaging‐based deep learning have helped connect fungal colonization phenotypes with soil phosphorus dynamics and microbial functional regulation (Evangelisti et al. 2021; Zhu et al. 2025). These approaches can therefore broaden the evidence base around enriched GO terms by identifying candidate functions, linking genes to prior knowledge, and prioritizing mechanisms for further investigation (Ahi and Karagic 2026). However, AI‐assisted inference should not be treated as a bias‐free alternative to GO enrichment. Many models depend on literature, sequence databases, curated annotations, or expression compendia that are themselves shaped by uneven annotation depth, model‐organism bias, and overrepresentation of well‐studied genes and taxa (Haynes et al. 2018; Oziolor et al. 2021; B. Xue and Rhee 2023; Phan et al. 2025). As a result, predictions for non‐model ecological taxa may remain biased toward conserved or heavily annotated functions, while lineage‐specific, habitat‐specific, or poorly characterized mechanisms may be missed (Primmer et al. 2013; Freedman and Sackton 2024; Vandepoele et al. 2024). In addition, deep learning predictions may be difficult to interpret mechanistically, creating a risk that opaque model outputs simply replace broad GO terms as a new endpoint of inference. For ecological omics, the value of AI‐assisted methods lies not in replacing curated annotation, lineage‐aware comparison, environmental contextualization, or experimental validation, but in helping to organize and prioritize evidence around these complementary approaches. Used critically, these approaches can support annotation refinement, candidate‐gene prioritization, literature synthesis, and hypothesis generation, thereby helping move interpretation from broad functional labels toward more focused, testable ecological mechanisms.

### 6.2 Context‐Aware and Lineage‐Aware Interpretation

#### 6.2.1 Environment‐Aware Functional Annotation

One way to improve ecological specificity is to integrate GO analysis with environmental metadata using tools and ontologies that explicitly connect molecular function with habitat variables (Blumberg et al. 2022; Buttigieg et al. 2016; L. Richardson et al. 2023). The Environment Ontology (EnvO) provides controlled vocabulary for describing environmental contexts, while community platforms such as MGnify integrate harmonized meta‐omics and sample metadata. Dedicated resources such as Ocean Gene Atlas (OGA2) further allow users to examine how gene distributions co‐vary with environmental features across ecological gradients (Blumberg et al. 2022; L. Richardson et al. 2023; Vernette et al. 2022). For example, in marine microbial assemblages, OGA2 can be used to relate gene abundance and co‐variation patterns with temperature, oxygen availability, nutrient concentrations, and seasonal water‐column structure (Vernette et al. 2022). Such approaches help place enriched GO terms within measurable environmental conditions rather than treating them as isolated functional labels. In practice, integrating GO enrichment results with environmental metadata can help distinguish ecologically meaningful responses from broad cellular maintenance programs that recur across many systems (Eloe‐Fadrosh et al. 2024). Although environmental association alone does not establish causality, it provides an important layer of ecological context that can substantially improve interpretation compared with enrichment statistics alone.

#### 6.2.2 Species‐Specific and Phylogenetically Informed Annotation Pipelines

For non‐model organisms, standard GO‐based annotations may lack specificity when they rely heavily on homology with distantly related, well‐characterized species. Phylogenetically informed annotation pipelines can improve functional interpretation by prioritizing information from closely related taxa, lineage‐specific gene families, and environmentally relevant reference datasets. Tools such as eggNOG‐mapper v2, when combined with custom databases, can refine functional assignments compared with generic annotation workflows (Cantalapiedra et al. 2021). For instance, comparative studies of saprotrophic and ectomycorrhizal fungi have revealed lineage‐specific expansions and contractions in hydrolases, transporters, and small secreted proteins, providing ecological resolution beyond broad GO categories (Harder et al. 2024; Looney et al. 2022; Miyauchi et al. 2020). Phylogenetically informed approaches can also reduce annotation‐transfer errors arising from homology‐based transfer across distantly related taxa (De Crécy‐Lagard et al. 2022), which is particularly important in groups characterized by horizontal gene transfer or genome duplication, where misannotation may be common (Goldfarb et al. 2025; Hämälä et al. 2024; Mariault et al. 2025). By grounding functional prediction in evolutionary context, these approaches can help distinguish lineage‐specific adaptations associated with local ecological conditions from broadly conserved cellular functions (De Crécy‐Lagard et al. 2022; Hämälä et al. 2024). Although they remain dependent on available comparative data and the accuracy of evolutionary inference, they provide a practical route for improving ecological specificity and reducing the semantic flattening that often accompanies generalized GO interpretation.

#### 6.2.3 Gene‐To‐Phenotype Mapping

Gene–environment association (GEA) approaches help connect allelic variation with environmental gradients such as climate, soil chemistry, hydrology, thereby translating broad GO categories into more specific hypotheses about environmental adaptation. Current best practices emphasize rigorous sampling design, correction for spatial autocorrelation and population structure, and the use of complementary approaches such as redundancy analysis (RDA) and latent factor mixed models (LFMM), which can help detect weak, polygenic signals typical of natural populations (Forester et al. 2018; Lasky et al. 2023). Once candidate loci or co‐expression modules are identified, expression quantitative trait locus (eQTL) mapping can place them within a regulatory context by revealing tissue specificity and the direction or magnitude of regulatory effects. Co‐expression networks can further provide module‐level information about coordinated biological processes, helping move interpretation beyond term‐level enrichment toward mechanistic hypotheses involving candidate regulators and targets (Shu et al. 2024). Transcriptome‐wide association studies (TWAS) extend this framework by integrating genome‐wide association data with expression reference panels to identify genes whose genetically regulated expression is associated with phenotypic variation (B. Li and Ritchie 2021). Together, GEA, eQTL, and TWAS approaches provide a layered framework for evaluating whether enriched GO categories are genuinely associated with ecological traits or environmental pressures. Rather than treating enrichment as an endpoint, these methods help cross‐validate GO signals against phenotypes, environments, and regulatory architecture, thereby prioritizing candidate genes and pathways for downstream validation and reducing the risk of mistaking statistical association for ecological mechanism.

#### 6.2.4 Temporal, Multi‐Omic, Spatial, Activity‐Based and Experimental Evidence

Rather than serving as retrospective examples of failed interpretation, the approaches discussed below are more constructively viewed as ways of refining broad functional categories into more mechanistically grounded ecological insight when additional layers of evidence are available.

#### 6.2.5 Multi‐Omics Convergence and Temporal Modelling

Ecological function is inherently dynamic rather than static, whereas GO enrichment typically captures only a single snapshot of biological activity (Oh and Li 2021). Integrating transcriptomics with proteomics, metabolomics, or lipidomics, epigenetic or epitranscriptomic information, and other regulatory RNA layers can help track how functional responses emerge, shift, and stabilize through time (Frese et al. 2024; Pashay and House 2026; Zhang et al. 2024). Temporal modelling approaches, including dynamic Bayesian networks and trajectory inference, further allow reconstruction of regulatory cascades and separation of early signalling responses from longer‐term acclimation or adaptation processes (Marku and Pancaldi 2023; Suter et al. 2022; Van den Berge et al. 2020). For example, studies of seasonal plant dynamics (Yumoto et al. 2024) and during amphibian metamorphosis (Zhang et al. 2024) have used such approaches to distinguish transient from sustained developmental or adaptive programs. Mapping enriched GO categories onto time‐structured networks can therefore help identify which pathways are activated early, which persist over time, and which regulators may coordinate these transitions (Marku and Pancaldi 2023; Oh and Li 2021; Suter et al. 2022; Van den Berge et al. 2020; Yumoto et al. 2024). Although temporal and multi‐omic datasets are more demanding in terms of sampling and analytical complexity, they provide an important route for moving beyond static enrichment summaries toward mechanistic understanding of ecological responses.

#### 6.2.6 Stable‐Isotope Probing and Metaproteomics

GO enrichment alone generally cannot determine which organisms within a community are actively performing a given function. Quantitative stable‐isotope probing (qSIP), which combines ^13^C‐ or ^15^N‐labelled substrates with isopycnic gradients, directly identifies active taxa and their substrate assimilation under environmentally realistic conditions. Recent methodological developments and high‐throughput pipelines have made qSIP increasingly feasible at ecological scales (Nuccio et al. 2022; Vyshenska et al. 2023). When combined with genome‐resolved metagenomics, qSIP can link metabolic activity to taxonomic identity and functional potential, helping clarify how environmental changes such as precipitation shifts or resource pulses select for particular traits and organisms (Greenlon et al. 2022). Metaproteomics provides a complementary perspective by identifying proteins that are produced within environmental samples. Advances in workflow standardization, quality‐control procedures, and mass spectrometry pipelines have substantially improved reproducibility across soils, aquatic systems, and biofilms (Armengaud 2023; Nebauer et al. 2024). Together, qSIP and metaproteomics move interpretation beyond broad categories such as “carbohydrate metabolism” toward identification of the specific taxa, enzymes, and pathways actively driving ecological processes under particular environmental conditions. In this way, they provide a practical means of testing whether GO‐enriched processes reflect generic cellular maintenance or ecologically relevant activity.

#### 6.2.7 Single‐Cell and Spatial Omics

Bulk omics combined with GO enrichment generally cannot localize function to specific cell types, tissues, or spatial contexts. Single‐cell and spatial omics approaches address this limitation by revealing where and when enriched processes occur and which cells are responsible for them. In plants, methods such as PHYTOMap enable transgene‐free multiplexed fluorescence in situ hybridization across whole tissues, allowing researchers to map modules associated with GO terms such as “cell wall biogenesis” or “ROS response”, onto particular cell layers and developmental stages (Nobori et al. 2023). Similarly, single‐cell atlases in corals have identified more than 40 cell types and revealed stage‐specific calcification and immune programs, thereby clarifying which cell types contribute to the processes represented by GO categories such as “biomineralization” or “response to stress” during warming or disease exposure (Levy et al. 2021). More broadly, recent methodological work has provided practical guidance for applying single‐cell and spatial methods to ecological questions involving stress responses, symbioses, and host–microbe interactions (Nobori 2025). By anchoring enriched GO categories to cell identity, position, and developmental trajectory, these methods substantially improve the biological resolution of interpretations in ecological omics.

#### 6.2.8 Synthetic Communities and Genome Editing

When causality is required, defined synthetic communities (SynComs) allow researchers to perturb community composition or host signalling and observe mechanistic outcomes that GO alone cannot resolve. A 35‐member Arabidopsis SynCom suppressed host immunity in the community context (not evident from single‐strain assays), revealing how commensals rewire pattern‐triggered responses (Teixeira et al. 2021). Specialized metabolites can be tested directly: coumarins reshape root community composition via a redox mechanism under Fe limitation, shifting specific Pseudomonas taxa and host phenotypes (Voges et al. 2019); reproducible, tunable soil SynComs now enable cross‐lab tests and trait gains beyond correlative omics (Coker et al. 2022). Where feasible, genome editing closes the loop at the gene level: CRISPR/Cas9 is established in a reef‐building coral (Cleves et al. 2018), and targeted HSF1 knockout reduced larval heat tolerance, providing a rare, direct causal link from a GO‐implicated stress regulator to ecological resilience (Cleves et al. 2020); broader cnidarian toolkits (Hydractinia knock‐ins; Clytia CRISPR) show growing generality for functional tests in non‐models (Momose et al. 2018; Sanders et al. 2018). Importantly, these approaches are not intended to replace GO enrichment, but to provide downstream validation and mechanistic refinement where feasible. Used alongside enrichment analysis, co‐expression networks, and environmental context, they help distinguish generic stress signatures from the genes, pathways, interactions, and organisms that actively drive ecological responses.

## 7 Conclusion

Omics has transformed ecology, yet the field too often treats GO enrichment as though statistical association were equivalent to biological explanation. Enrichment can organize signal, but it cannot, on its own, resolve mechanism, context or causality. Enrichment can organize biological signals and, for some descriptive or comparative questions, may provide an adequate summary. However, it cannot, on its own, resolve mechanism, context, or causality when such claims are the intended endpoint. When used as an endpoint, it encourages familiar, generic narratives and hinders the development of precise, testable ecological hypotheses. In this manuscript, we propose GO‐Deep, a framework that treats enrichment not as a destination but as a disciplined starting point for deeper inference, as illustrated conceptually in Figure 1A. The GO‐Deep approach, treating enrichment as a starting point for layered inference, is laid out stepwise in Table 4. Building on the strategy classes summarized in Table 3, the GO‐Deep workflow in Table 4 should be read as a prioritization framework rather than a mandatory pipeline. Computationally accessible steps such as bias‐aware enrichment, redundancy control, annotation‐depth assessment. and environmental or lineage‐aware interpretation provide a minimal feasible version of GO‐Deep, whereas time‐series designs, multi‐omics, metaproteomics, stable‐isotope probing, synthetic communities, genome editing, and single‐cell or spatial assays provide deeper mechanistic or validation‐oriented evidence where feasible. These alternative implementation routes are summarized in Figure 1B. The central idea is straightforward: use GO enrichment as an entry point rather than a destination. Enriched categories should serve to nominate candidate processes, pathways, and genes that can then be interrogated through approaches that add biological structure and ecological context, including co‐expression and regulatory networks, environmental metadata integration, phylogenetically informed annotation, and time‐resolved or multi‐omics analyses. Under the GO‐Deep perspective, enriched GO terms become hypotheses to refine rather than conclusions to accept. Interpretation should therefore explicitly connect functional categories to ecological variables, environmental gradient, organismal traits, species interactions, and evolutionary context, while incorporating independent evidence whenever possible.

**FIGURE 1:**
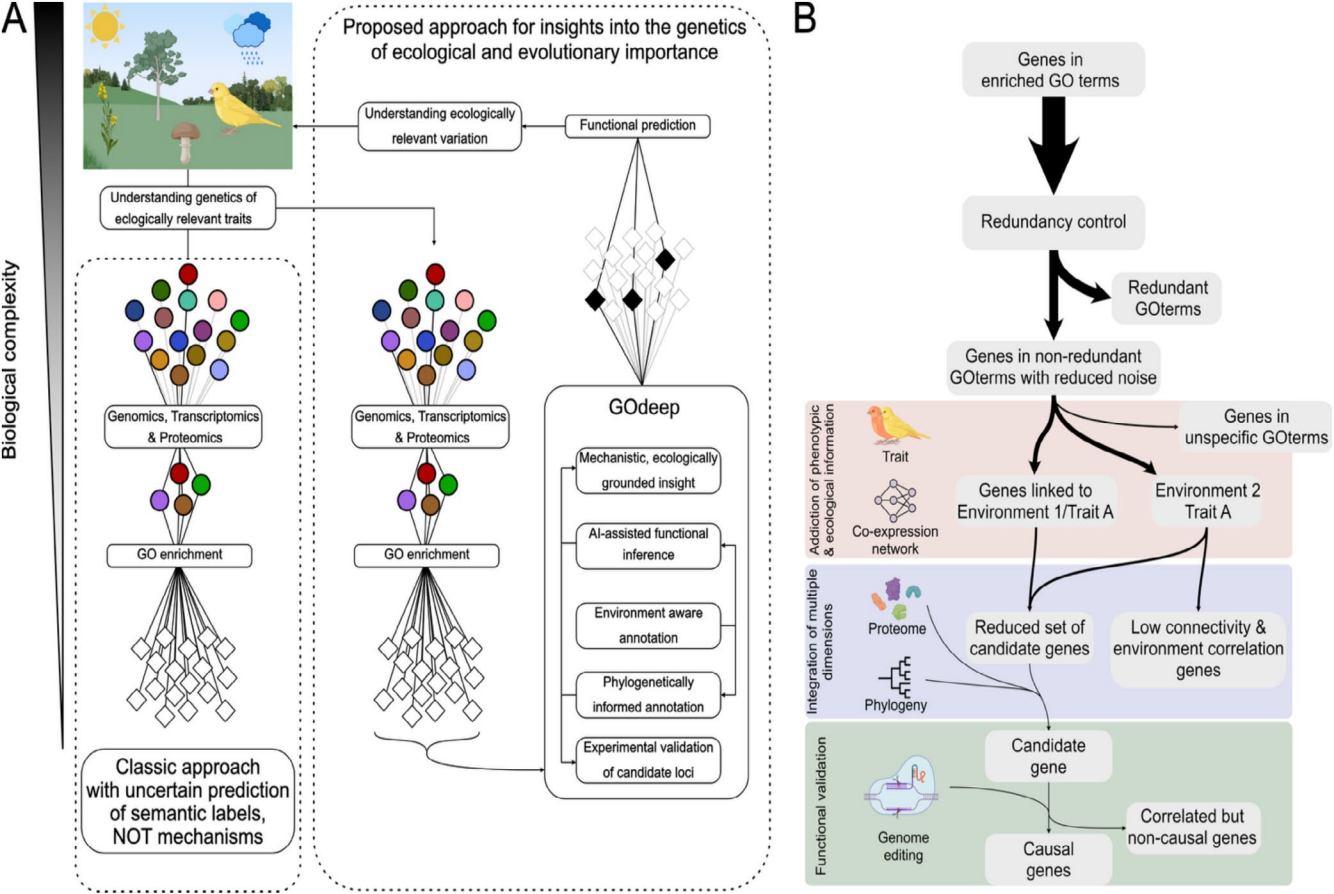
Current state of GO‐term analysis and a framework for moving forward. (A) Classic GO‐term often leads to semantic saturation, in which broad categories are repeatedly used to interpret Omics data. However, this rarely leads to new insights into ecologically relevant mechanisms of adaptation and evolution. We propose GO‐Deep, a novel framework that integrates molecular, phylogenetic, and experimentally validated data to build on classic GO‐term analysis rather than treating enriched GO‐terms as definite conclusions. Such an integrative framework will lead to more meaningful and ecologically relevant insights into the mechanistic basis of adaptation and evolution. Differently coloured circles depict different genes correlated with trait variation. Diamonds indicate GO‐terms associated with identified genes. Black diamonds indicate GO‐terms closely associated with trait variation after application of the GO‐Deep framework to ultimately come to meaningful inference of ecological relevance for genes from a knowledge‐based interactome. (B) Compact workflow showing minimal context‐aware (environment, trait, phylogeny aware) and validation‐oriented (e.g., Genome editing) application of GOdeep on multimodal data. Arrow size between grey boxes indicates number of genes. The GOdeep integration is depicted as “filtering” steps for genes: From a large number of correlated genes from large non‐specific semantic GOterms to a causal gene in an environmental context. Created in BioRender. Ahi and Karagic (2026) https://BioRender.com/ydpady4.

**TABLE 4:** GO‐Deep: A staged workflow for context‐aware functional inference.

GO‐Deep stage	What you do	What it adds	Output	Key references
1. Bias‐aware enrichment setup core	Define background, correct bias, robust stats	Prevents systematic false signals	Calibrated enrichment summary	Young et al. (2010), Timmons et al. (2015), Wijesooriya et al. (2022), Ziemann et al. (2024)
2. Redundancy control core	Trim/Cluster/Filter similar GO terms	Reduces semantic noise	Concise, interpretable term set	Jantzen et al. (2011), Gu and Hübschmann (2023), Ozisik et al. (2022), Wang et al. (2020)
3. Network placement (When supported by replication/Data structure)	Overlay genes on co‐expression/PPI modules	Structure + candidate regulators	Modules/Hubs tied to signal	Morabito et al. (2023), Ovens et al. (2021), Szklarczyk et al. (2023), Kotlyar et al. (2022)
4. Environment or trait linkage core (when metadata are available)	Bind modules/Genes to habitat variables (EnvO/Metadata)	Ecological specificity	Gene–environment covariation	Buttigieg et al. (2016), Vernette et al. (2022), Richardson et al. (2023), Cernava et al. (2022)
5. Lineage‐aware annotation check core for non‐model taxa	Orthology/Phylogeny/Custom DB annotation	Reduces transfer errors	Refined functional calls	Cantalapiedra et al. (2021), De Crécy‐Lagard et al. (2022), Vandepoele et al. (2024), Oziolor et al. (2021)
6. Time/Trajectory structure (optional extension)	Time‐series/Pseudotime/DBNs	Directionality + phases	Early vs. late response chains	Oh and Li (2021), Van den Berge et al. (2020), Suter et al. (2022), Marku and Pancaldi (2023)
7. Multi‐omic/Phenotype anchoring (optional extension)	Integrate proteome/Metabolome/Traits	Triangulation toward mechanism	Convergent causal candidates	Ryan et al. (2025), Frese et al. (2024), Zhang et al. (2024), Li et al. (2024)
8. Causal validation (when feasible)	SynComs, editing, activity assays	Moves beyond labels	Confirmed drivers vs. correlates	Teixeira et al. (2021), Cleves et al. 2018, Cleves et al. (2020), Nuccio et al. (2022), Armengaud (2023)

*Note:* A stepwise framework that reframes GO enrichment as an entry point rather than a conclusion, with stages prioritized according to data availability, organismal tractability, metadata quality, and feasibility of validation. Stages are ordered from calibration and redundancy control to network placement, environment/lineage linkage, temporal structuring, multi‐omic triangulation, and (where feasible) causal validation. Each stage specifies the added evidence type and expected output, with references to representative tools, standards, and benchmarking literature.

Progress will also depend on broader changes in analytical practice and scientific culture. Reviewers and journals should evaluate not only whether enrichment analyses are statistically correct, but also whether the resulting biological claims are justified by the available evidence. Training in ecological omics should emphasize critical, context‐aware interpretation, encouraging researchers to move beyond descriptive labels toward mechanistic reasoning. At the same time, continued community efforts to improve functional annotation for underrepresented taxa and to expand environment‐linked ontologies will help reduce dependence on broad, catch‐all categories. Ultimately, the success of ecological omics should not be judged by the number of enriched GO terms reported, but by whether studies improve understanding of how systems function and respond to change. This includes identifying regulatory innovations, linking genes and pathways to traits and environments, and connecting molecular processes to population‐ and ecosystem‐level dynamics. Used in this way, GO enrichment remains highly valuable, not as a shortcut to explanation, but as a structured point of departure for more mechanistic and ecologically grounded inquiry.

## Author Contributions

E.P.A. and S.P. conceived the idea for this manuscript. E.P.A. and S.P. conducted the literature review and drafted the initial version, N.K. made the figure, and all authors collaboratively revised and finalized the manuscript.

## Funding

The authors have nothing to report.

## Ethics Statement

This article is an Opinion manuscript and does not report new experiments involving humans, animals, field sampling, or the collection of biological material. Therefore, ethical approval was not required.

## Conflicts of Interest

The authors declare no conflicts of interest.

## Data Availability

Data sharing not applicable to this article as no datasets were generated or analysed during the current study.
